# All-Ceramic Single Crown Restauration of Zirconia Oral Implants and Its Influence on Fracture Resistance: An Investigation in the Artificial Mouth

**DOI:** 10.3390/ma8041577

**Published:** 2015-04-01

**Authors:** Ralf-Joachim Kohal, Jolanta Bernadette Kilian, Susanne Stampf, Benedikt Christopher Spies

**Affiliations:** 1 Department of Prosthetic Dentistry, Center for Dental Medicine, University Hospital Freiburg, Albert-Ludwigs-University, Freiburg 79106, Germany; E-Mail: ralf.kohal@uniklinik-freiburg.de; 2 Private Practice, Singen 78224, Germany; E-Mail: jolanta.kilian@uniklinik-freiburg.de; 3 Department for Medical Biometry and Medical Informatics, Institute for Medical Biometry and Statistics, Albert-Ludwigs-University, Freiburg 79104, Germany; E-Mail: Susanne.Stampf@usb.ch

**Keywords:** zirconia implants, Y-TZP, fracture load, bending moment, artificial ageing

## Abstract

The aim of the current investigation was to evaluate the fracture resistance of one-piece zirconia oral implants with and without all-ceramic incisor crowns after long-term thermomechanical cycling. A total of 48 implants were evaluated. The groups with crowns (C, 24 samples) and without crowns (N, 24 samples) were subdivided according to the loading protocol, resulting in three groups of 8 samples each: Group “0” was not exposed to cyclic loading, whereas groups “5” and “10” were loaded with 5 and 10 million chewing cycles, respectively. This resulted in 6 different groups: C0/N0, C5/N5 and C10/N10. Subsequently, all 48 implants were statically loaded to fracture and bending moments were calculated. All implants survived the artificial aging. For the static loading the following average bending moments were calculated: C0: 326 Ncm; C5: 339 Ncm; C10: 369 Ncm; N0: 339 Ncm; N5: 398 Ncm and N10: 355 Ncm. To a certain extent, thermomechanical cycling resulted in an increase of fracture resistance which did not prove to be statistically significant. Regarding its fracture resistance, the evaluated ceramic implant system made of Y-TZP seems to be able to resist physiological chewing forces long-term. Restauration with all-ceramic single crowns showed no negative influence on fracture resistance.

## 1. Introduction

The clinical long-term results reported for oral implants made of titanium and its biomedical alloys [1,2] have made titanium the “gold standard” material for their fabrication. Besides its favorable physical and mechanical properties [3], titanium shows a high biocompatibility and a low potential of corrosion [4]. Nevertheless, there have been some concerns that titanium might evoke an unwelcome host reaction but the significance of titanium as a cause of allergic reactions in patients with dental implants remains unproven [5,6,7,8,9]. Notwithstanding, the rising popularity of metal-free reconstructions motivates clinicians to offer an implant made of another material than titanium. Its predominant biomechanical behavior among biomedical ceramics makes zirconium dioxide to be the ceramic of choice for the fabrication of dental implants [10]. It has been described that zirconium dioxide exhibits no cytotoxic, sensitizing, mutagenic or oncogenic effect [11,12,13,14,15,16,17,18]. Primarily, Yttria-stabilized zirconia (Yttria-stabilized tetragonal zirconia polycrystal = Y-TZP) seems to be the favorable core material for the manufacturing of dental implants. This material is characterized by a dense, monocrystalline homogeneity, possesses a low thermal conductivity, a low corrosion potential and a good radiopacity [19]. Y-TZP exhibits high flexural strength values (900–1200 MPa) and fracture toughness (9–10 MPa m^0.5^) owing to a phase transformation toughening mechanism [20]. Nevertheless, aqueous induced dissolution and phase changes of zirconia ceramics resulting in mechanical strength degradation (also known as low-temperature degradation; LTD) are a major concern regarding the clinical long-term performance of Y-TZP [21]. The stability and durability of prepared and unprepared one-piece dental implants made of Y-TZP after up to 20 years of masticatory simulation seems to range within the limits of clinical acceptance [22,23,24]. Regrettably, none of the mentioned investigations evaluated the influence of a restoration on the fracture-resistance of zirconia oral implants, which resembles clinical reality. Currently available literature for zirconia oral implants with single crown restorations is limited to experimental implant systems over a simulated time period of 5 years with an exerted load of 45–50 N [25,26]. Therefore, the purpose of the current investigation was to evaluate the thermomechanical stability of a market-ready one-piece zirconia implant system with and without all-ceramic single crown reconstructions before and after artificial loading conditions with an exerted load of 98 N in an aqueous environment over a simulated time period of 20 and 40 years, respectively. Non-loaded samples served as control.

## 2. Materials and Methods

### 2.1. Grouping and Sample Preparation

**Figure 1:**
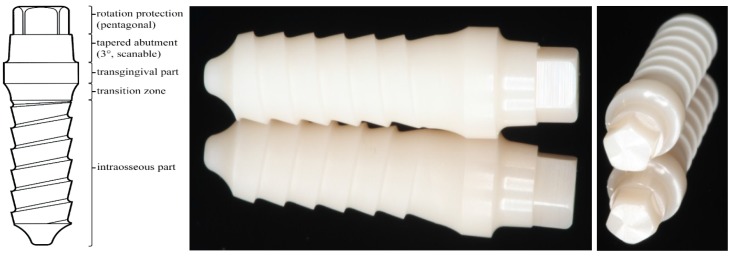
The evaluated implant system (zit-z, Ziterion GmbH, Uffenheim, Germany) and its metrics used for the experiment: 4.0 mm diameter, 13 mm intraosseous length, 2.5 mm transgingival height, 4 mm abutment height (pentagonal and tapered part).

A total of 48 “zit-z” zirconia dental implants (4.0 mm diameter, 13 mm intraosseous length, 2.5 mm transgingival height, 4 mm abutment height; Ziterion GmbH, Uffenheim, Germany) were used for the experiment (Figure 1). Material properties, as indicated by the manufacturing company and according to ISO 13356, are shown in Table 1. The implants were equally divided into two groups (Table 2). Group C consisted of 24 implants with all-ceramic (IPS e.max^®^ CAD; Ivoclar Vivadent, Schaan, Liechtenstein) incisor single crown restorations. The 24 implants of group N were used “as delivered”. Both groups were further divided into three subgroups: subgroup “0”—eight implants that were not subjected to artificial loading in the chewing machine; subgroup “5”—eight implants that were subjected to 5 million loading cycles in an aqueous environment in the artificial mouth and subgroup “10”—eight implants that were subjected to 10 million loading cycles in an aqueous environment in the artificial mouth. In the end 6 different groups for evaluation resulted: C0, C5, C10, N0, N5 and N10.

**Table 1:** Material properties according to the manufacturer.

Characteristics	Unit	Y-TZP
Components		ZrO_2_/Y_2_O_3_
Composition	wt%	95/5
Density	g/cm^3^	>6.0
Grain size	µm	<0.6
Bending strength	MPa	>1200

**Table 2:** Grouping of test and control specimens.

48 Zirconia Implants					
Group “C”			Group “N”		
n = 24			n = 24		
Implants with single crown restauration			Implants without restauration (“as delivered”)		
C0	C5	C10	N0	N5	N10
n = 8	n = 8	n = 8	n = 8	n = 8	n = 8
0 cycles	5 × 10^6^ cycles	10 × 10^6^ cycles	0 cycles	5 × 10^6^ cycles	10 × 10^6^ cycles
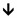	Dynamic loading	Dynamic loading	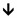	Dynamic loading	Dynamic loading
Static loading test					

**Figure 2:**
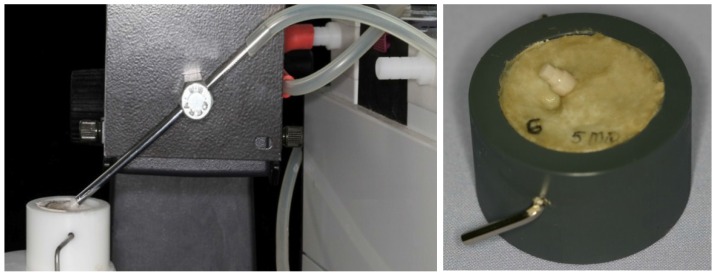
Embedding of implants at an angle of 45° to the vertical with the help of a vacuum pump to fix the samples in place.

All implants were embedded in an autopolymerizing acrylic resin (Technovit^®^ 4000, Heraeus Kulzer, Wehrheim, Germany) into special sample holders at an angle of 45° to the vertical (Figure 2), replicating the position of upper central incisors [27]. In order to represent a physiological clinical situation after one year [28,29], the implants were embedded 0.5 to 1 mm above bone level. The resin had a modulus of elasticity of approximately 12 GPa which approximates that of human bone (10–18 GPa) [30].

**Figure 3:**
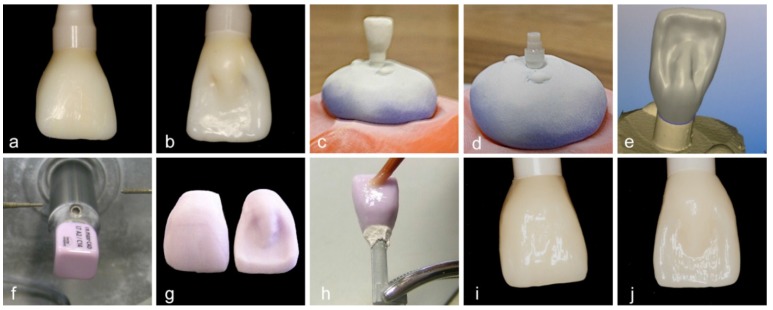
Manufacturing process of the incisor single crowns: Wax-up to implant abutment (**a**/**b**); Scan of Wax-up (**c**) and abutment (**d**); Computer-aided design (**e**); Computer-aided manufacturing (**f**) and restoration before glazing (**g**); Glazing (**h**); Final restoration (**i**/**j**).

**Figure 4:**
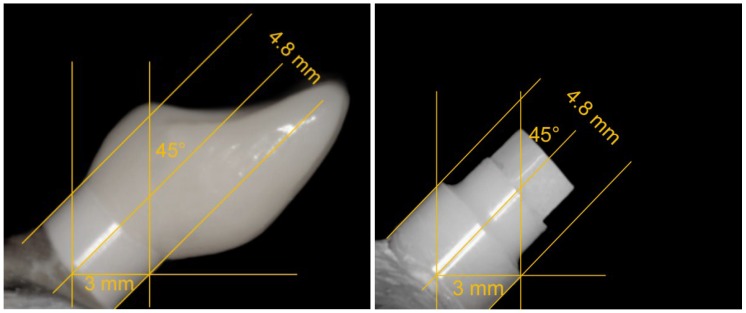
Standardized photographs of the embedded implants with (**left**; groups C) and without (**right**; groups N) restoration allowed the measurement of the lever arm (the diameter of the transgingival platform served as reference).

A wax-up of a central incisor to an implant abutment has been performed (Figure 3a,b). After a scan (inEos, Sirona, Bensheim, Germany) of the bare abutment and the wax-up (Figure 3c/d), 24 monolithic single crowns were finally modified via computer-aided design (Figure 3e) and subsequently grinded out of pre-sintered lithium disilicate blanks (IPS e.max^®^ CAD; Figure 3f/g) by computer-aided manufacturing (inLab 3D, Sirona), followed by a final sintering process (30 min, 850 °C; Figure 3g–j). According to manufacturer’s instructions for bonding to zirconia, the polished lithium disilicate crowns were adhesively cemented (Multilink Automix, Ivoclar Vivadent) to the abutments of group “C” implants and finally light-cured with a polymerization lamp. Standardized photographs were used to measure the lever arm for each of the 48 samples (Figure 4).

### 2.2. Dynamic Loading Test

**Figure 5:**
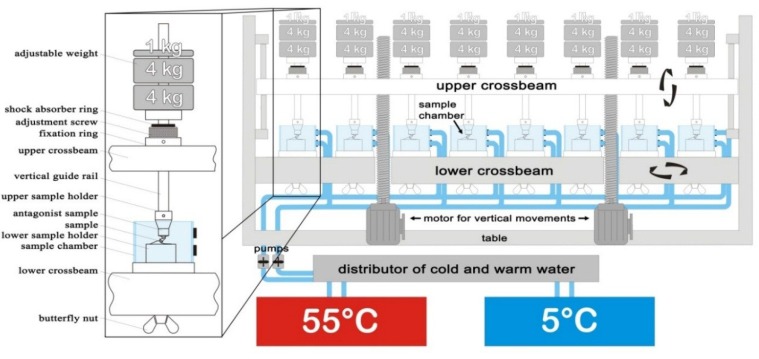
Schematic drawing of the chewing simulator (Willytec, Munich, Germany). The vertical guide rail and the sample holder weigh another 1 kg.

Thirty-two of the specimens were thermomechanically aged in a computer-controlled dual axis-chewing simulator in an aqueous environment (Willytec, Munich, Germany; Figure 5) in order to simulate twenty years (5 million cycles; subgroups C5/N5) and almost forty years (10 million cycles; subgroups C10/N10) of clinical service, assuming an annual masticatory performance of 240,000–250,000 occlusal contacts [31]. The chewing simulator-environment consisted of eight identical sample chambers filled with water, two stepper motors controlling vertical and horizontal movements of the antagonists (Steatit^®^ ceramic balls, 6 mm in diameter, Hoechst Ceram Tec, Wunsiedel, Germany) against the implant samples, and a hot and cold water circulation system (Haake, Karlsruhe, Germany). The antagonist ball had a Vickers hardness similar to that of enamel [32]. The applied load in the chewing simulator was 98 N (10 kg) [33,34] and the point of load application on the implants was placed on the palatal of the single crowns (groups “C”) and the upper edge of the implant abutments (groups “N”), respectively. The load was applied onto the implants by combined vertical (6 mm) and horizontal (0.5 mm) movements, which—via computerized adaptation—represented an approximation to the physiological masticatory cycle of axial pressure and horizontal shear. The cyclic loading was set at 1.6 Hz. The thermocycling was from 5 °C to 55 °C for 60 s each with an intermediate pause of 12 s, maintained by the thermostatically-controlled liquid circulator (Haake, Karlsruhe, Germany). During the dynamic loading, all samples were examined twice a day. The chewing machine needed approximately 36 and 72 days to accomplish 5 and 10 million cycles, respectively. Fractures of the implants were recorded as a failure. The details of the settings of the chewing simulator machine are listed in Table 3.

**Table 3:** Settings of the chewing simulator machine.

Chewing Cycles	5,000,000/10,000,000
Cycle frequency	1.6 Hz
Vertical movement	6 mm
Horizontal movement	0.5 mm
Descending speed	60 mm/s
Rising speed	55 mm/s
Forward speed	60 mm/s
Backward speed	55 mm/s
Applied weight per sample	10 kg (98 N)
Hot dwell time	60 s
Hot bath temperature	55 °C
Cold dwell time	60 s
Cold bath temperature	5 °C
Intermediate pause	12 s

### 2.3. Static Loading Test

All samples that survived the exposure to the chewing simulator were statically loaded using a universal-testing machine (Zwick, Z010/TN2S, Ulm, Germany) until fracture occurred. All samples were loaded at the same contact point used for the dynamic loading. A vertical compressive load was applied on the palatal side of the angulated implants under a crosshead speed of 10 mm/min. The loads required for fracturing the samples were recorded using the X-Y writer of the Zwick testXpert^®^ V 7.1 software, with failure recorded at the first sharp drop-down of the graphical curve. The recorded data were automatically analyzed and a graph was drawn for each sample.

### 2.4. Statistical Analysis

A linear model was fitted to evaluate the effects of restoration type (C = single crown, N = no restoration) and loading protocol (0, 5 and 10 million chewing cycles) on the response variables (fracture load and bending moment). This analysis was performed separately for each response variable. Least-square means with 95% confidence intervals were derived from these models. Thus, the continuous response variables were modelled as a function of restoration type, loading protocol and their interaction. Overall tests for main and interaction were done as well as pairwise comparisons between the levels of the explanatory variables and interaction. Therefore, the method of Tukey-Kramer was applied to adjust for multiple testing. The level of significance was set to 0.05. Differences of least-square means with corresponding 95% confidence intervals were plotted. Furthermore, boxplots were used for the graphical presentation of the data. All computations were performed with the statistical software SAS (SAS system v9.1.2; SAS Institute Inc., Cary, NC, USA) using PROC MIXED.

## 3. Results

### 3.1. Dynamic Loading Test

All 24 artificially loaded implants survived the dynamic loading test resulting in 100% survival. However, five single crowns of group C10 had to be re-cemented during the dynamic loading test. One crown of group C10 fractured after 4.5 million chewing cycles and had to be replaced. Therefore, the survival rate for the restoration was 100% for group C5 and 87.5% for group C10. All single crowns of group C5/C10 showed a distinct wear of the palatal contact point. Regarding the loading protocol as well as the restoration type, the lever arms showed no major alterations (2.9–3.0 mm). Therefore, the exerted bending moment on the samples during the dynamic loading test was roughly equivalent for all samples (28.4–29.4 Ncm).

### 3.2. Static Loading Test

**Figure 6:**
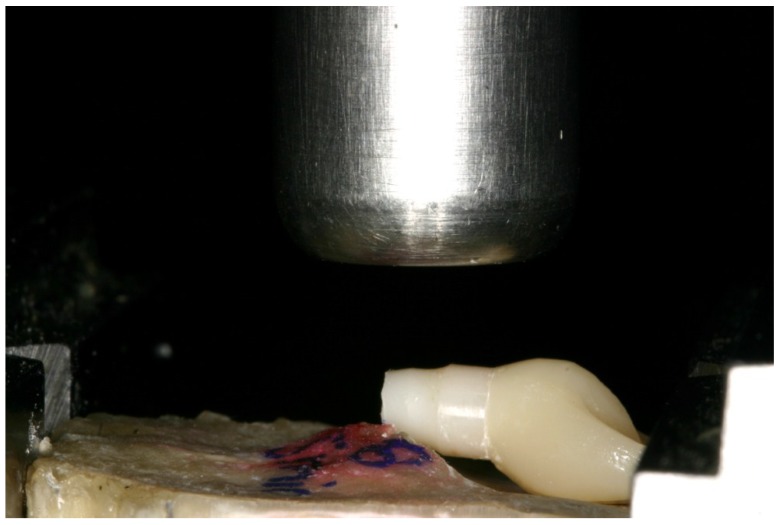
Fracture of all samples occurred between the first and the second thread slightly below the embedding margin.

**Figure 7:**
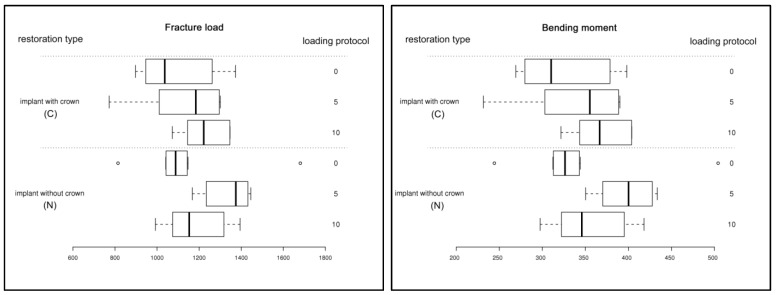
Box plot diagrams of the fracture load in [N] (**left**) and the bending moment in [Ncm] (**right**) sorted by restoration type (C: implant with crown; N: implant without crown) and loading protocol (0, 5 and 10 million chewing cycles).

The pattern of fracture for the implants is shown in Figure 6: Independent of the presence of a restoration, all implants fractured slightly below the embedding margin between the first and the second thread. The mean values of the fracture load and bending moment are listed in Table 4. Furthermore, the calculated values of the fracture load and the bending moment were illustrated in the form of box plots (Figure 7).

**Table 4:** Means of measurements (fracture load, bending moment) for the different test groups (n = number of specimens).

Subgroup	*n*	Fracture load [N] F		Bending moment [Ncm] M	
		mean	*SD*	mean	*SD*
C0	8	1095.2	*183.4*	325.6	*53.3*
C5	8	1131.5	*189.1*	339.5	*56.7*
C10	8	1230.6	*109.7*	369.2	*32.9*
N0	8	1130.5	*245.6*	339.2	*73.7*
N5	8	1336.6	*110.4*	397.5	*32.1*
N10	8	1184.4	*147.0*	355.3	*44.1*

### 3.3. Statistical Analysis

For the bending moment and the fracture load, the overall test revealed no statistically significant differences regarding the restoration type (C: with single crown restoration; N: no restoration), loading protocol (0, 5 and 10 million chewing cycles) and their interaction. Furthermore, pairwise comparisons between the levels of the explanatory variables and interaction showed no significance after adjustment (Tukey-Kramer; Table 5).

**Table 5:** Pairwise comparison (adjustment: Tukey-Kramer) of subgroups regarding their response variables (bending moment and fracture load).

Comparison	Bending moment		Fracture load	
	adj. *p*-value	Significance	adj. *p*-value	Significance
C0 : C5	0.994	not significant	0.9981	not significant
C0 : C10	0.5322	not significant	0.6137	not significant
C0 : N0	0.9945	not significant	0.9983	not significant
C0 : N5	0.0728	not significant	0.0732	not significant
C0 : N10	0.8497	not significant	0.9001	not significant
C5 : C10	0.8493	not significant	0.8537	not significant
C5 : N0	1.0	not significant	1.0	not significant
C5 : N5	0.2239	not significant	0.1802	not significant
C5 : N10	0.9886	not significant	0.989	not significant
C10 : N0	0.8439	not significant	0.8485	not significant
C10 : N5	0.8727	not significant	0.8146	not significant
C10 : N10	0.9939	not significant	0.9942	not significant
N0 : N5	0.2192	not significant	0.1761	not significant
N0 : N10	0.9876	not significant	0.9881	not significant
N5 : N10	0.5658	not significant	0.4894	not significant

## 4. Discussion and Conclusions

The mechanical properties concerning biomedical zirconia offered by the manufacturers and for instance required to satisfy ISO 13365 are solely based on mechanical strength values and aging kinetics measured on bending bars or discs, which is not simulating clinical reality and not considering any subsequent manufacturing procedures or surface modifications. However, it could be shown that such procedures are liable to compromise the long-term mechanical properties of zirconia or increase its sensitivity to ageing [35,36]. Therefore, long-term thermomechanical cycling in an aqueous environment of the market-ready product should be performed in advance to validate the functionality and safety of zirconia implants prior to their clinical use.

To simulate the above mentioned conditions to the extent possible, the experimental setup of the current investigation differed from ISO 14801, which does not include horizontal components of a dynamic loading or any environmental factors. Masticatory simulation trials should imitate the occlusal loading, create forces comparable to those which develop during horizontal and vertical components of masticatory motion and re-create environmental factors such as temperature and moisture fluctuations as found in the oral cavity (Krejci *et al.* 1994). ISO 14801 dictates the simulation of a 3 mm bone recession. According to clinical findings after 1 year [28], the implants of the current testing were embedded simulating 0.5–1 mm of bone recession. Therefore, the calculated pure fracture load values are not comparable to other investigations adapting ISO 14801. Nevertheless, the calculation of the bending moment includes the calculated lever arm in addition to the fracture load and is, therefore, more crucial and the significant value when comparing different investigations, respectively. The load applied to all implants during masticatory simulation was 98 N, and the direction of force between the implant and antagonist was set at 45° to the vertical. This force (98 N) was chosen to simulate the physiological loading of maxillary teeth, following a clinical investigation by Fontijn-Tekamp and coworkers who found normal chewing forces of 60 to 75 N in the anterior dentition, and 110 to 125 N in the posterior dentition [33].

In several in vivo evaluations, maximum bending moments of 27 Ncm have been measured at implant supported fixed partial dentures equipped with strain gauge abutments [37,38,39,40]. This value is comparable with the exerted bending moment during the dynamic loading test (28.4–29.4 Ncm). The mean bending moment values at the timepoint of fracture for all groups (326–398 Ncm) exceeded this value to the factor 10 or more. According to the mentioned investigations, the results of the current investigation support the use of the evaluated systems in clinical applications.

Explanation attempts for a transient increase in fracture resistance of Y-TZP after dynamic loading remain theoretical. Nevertheless, all measured deviations of fracture resistance in the current investigation proved to be not statistically significant. It might be a matter of time until the samples of the present investigation, which showed an increase in fracture resistance, reveal decreased fracture resistance due to a proceeding *t*➔*m* phase transformation. There are several in vitro investigations available in the literature focusing on the fracture resistance of zirconia oral implants with and without different modifications (e.g., preparation or restoration) after different loading protocols (e.g., 1.2 to 10 million chewing cycles, with or without thermocycling, 30-98 N exerted load) presenting increased as well as decreased fracture resistance values after the dynamic loading test [23,24,25,41,42]. Therefore, the process of zirconia’s aging kinetics seems to be not known exactly. Further research and more profound methodology (e.g., Raman spectroscopy and/or X-ray diffraction) after different loading protocols seem to be necessary.

Five restorations (all C10) showed de-cementations during the dynamic loading test after 5 million chewing cycles and had to be re-cemented once. None of the restorations of group C5/C10 had to be re-cemented before 5 million cycles. Since cement residues were solely on the restoration and not on the abutment, the zirconia/cement interface might be the weak point long-term. The “Metal/Zirconia Primer” was used for cementation according to manufacturer’s instructions. Therefore, further long-term investigations exceeding 20 years of masticatory simulation might be necessary to evaluate the optimal material properties and bonding procedures to achieve a more reliable connection between zirconia oral implants and their restorations. Nevertheless, re-cementations could be performed without complications and happened to a timepoint that is in the range of clinical acceptance.

The single crown restorations of the current investigation were grinded out of pre-sintered IPS e.max CAD blank (Ivoclar Vivadent), representing a lithium disilicate ceramic. One of eight crowns of group C10 fractured during the dynamic loading test, resulting in 87.5% survival for group C10. Regrettably, it might be a coincidence that this sample failed in group C10 and not in group C5 and, therefore, the presented survival rate might be misleading. Due to the failure before 5 million cycles the mentioned sample could have equally been assigned to group C5. Since the sample assignment to the different subgroups was random and before any testing procedure, this interrogation cannot be clarified afterwards. Furthermore, the ball bearing of the affected sample chamber had to be replaced due to deterioration induced malfunction shortly after the fracture occurred. Therefore, it cannot be ruled out that the failure was a result of temporarily uncontrolled forces.

Regarding its fracture resistance, the evaluated ceramic implant system made of Y-TZP seems to be able to resist physiological chewing forces long-term. Restauration with all-ceramic single crowns and different loading protocols in an aqueous environment showed no negative influence on fracture resistance.
